# Routine echocardiographic measurements in clinical practice: reproducibility of left and right ventricular indices and agreement with cardiac magnetic resonance

**DOI:** 10.3389/fcvm.2026.1843290

**Published:** 2026-08-05

**Authors:** Raul Eduardo Reyes-Toledo, David Gabriel David-Pardo, Gustavo Lemus-Barrios, Diego Rangel-Rivera, Claudia Jaimes, Yeisson Ávila-Cortés, Carlos Eduardo Guerrero-Chalela, Gabriel Salazar, Juan Felipe Vasquez-Rodriguez

**Affiliations:** 1 Non-Invasive Cardiovascular Diagnostic Methods, Fundación Cardioinfantil - Instituto de Cardiología, Bogotá, Colombia; 2 School of Medicine and Health Sciences, Universidad del Rosario, Bogotá, Colombia

**Keywords:** echocardiography, reproducibility, cardiac magnetic resonance imaging, interobserver variability, right ventricular function

## Abstract

**Background:**

Although the reproducibility of individual echocardiographic measurements has been previously investigated, evidence evaluating a comprehensive panel of routinely used left- and right-sided parameters within standardized real-world clinical workflows from Latin American centers remains limited. We aimed to evaluate the reproducibility of routine echocardiographic measurements, including intraobserver and interobserver variability, and to examine their agreement with cardiac magnetic resonance (CMR).

**Methods:**

Two cohorts of adult participants were included between 2022 and 2023: a clinical cohort consisting of patients referred for transthoracic echocardiography because of cardiovascular symptoms or routine cardiovascular evaluation, and a healthy reference group with no history of cardiovascular disease or self-reported systemic illness. Left- and right-sided echocardiographic measurements, including ventricular, atrial, and left ventricular outflow tract (LVOT) indices, were analyzed. CMR was used to assess left ventricular volumes and ejection fraction. Reproducibility was assessed by two echocardiographers using intraclass correlation coefficients and Bland–Altman analysis.

**Results:**

Sixty-three participants were included, 20 in the healthy reference group and 43 in the clinical cohort. Left ventricular measurements showed excellent reproducibility, whereas right-sided indices showed overall good reproducibility. LVOT diameter showed the lowest concordance. Compared with CMR, echocardiography underestimated left ventricular volumes, while agreement for left ventricular ejection fraction remained excellent.

**Conclusions:**

In routine clinical practice, standard echocardiographic measurements showed overall good reproducibility, with the strongest performance for left ventricular indices. Greater variability was observed for selected right ventricular measurements and LVOT diameter, whereas agreement with CMR was excellent for left ventricular ejection fraction despite underestimation of ventricular volumes.

## Graphical Abstract

**Figure:**
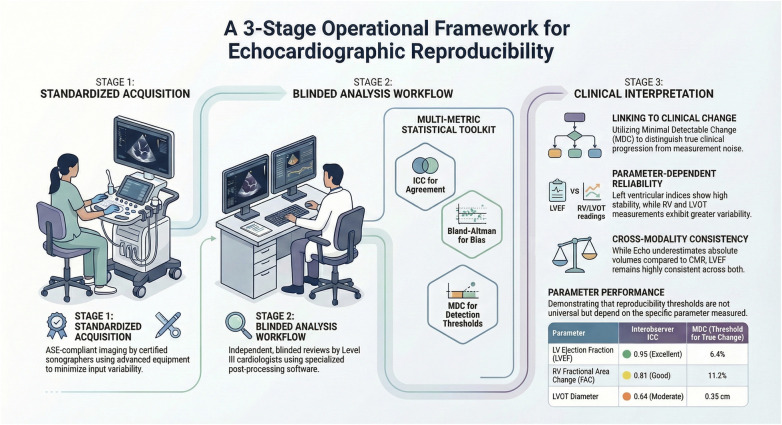

## Introduction

Echocardiography is the most widely used cardiovascular imaging modality in clinical practice because of its broad availability, relatively low cost, and ability to noninvasively assess cardiac structure and function, providing essential information for diagnosis, risk stratification, and therapeutic decision-making in a wide range of cardiovascular diseases (1).

Continuous quantitative variables obtained from a standard echocardiographic examination, such as cardiac chamber volumes, linear dimensions, and area measurements, constitute key parameters that influence a wide range of clinical decisions. These include the indication for devices such as implantable cardioverter-defibrillators, initiation of pharmacological therapies for heart failure, monitoring of chemotherapy-related cardiotoxicity, and determining the optimal timing for valvular interventions, among many other clinical applications (2).

The operator-dependent nature of echocardiography introduces a potential source of variability in these quantitative measurements. This variability may arise from several factors, including differences in image acquisition technique and rigor, delineation of endocardial borders, selection of the echocardiographic imaging planes analyzed, and the quality and technical characteristics of the ultrasound equipment used, and may even occur in repeated measurements performed by the same observers in the same patients (3).

For instance, several studies have demonstrated that left ventricular ejection fraction (LVEF), a parameter widely used in routine clinical practice, shows substantial variability in its measurement. These studies have highlighted that improvements in image quality through newer imaging technologies, rigorous standardization of acquisition protocols, and continuous training of personnel involved in echocardiographic analysis are essential to reduce measurement variability and enable reliable longitudinal assessment over time (4, 5).

Despite the clinical relevance of this issue, the available evidence regarding the reproducibility of echocardiographic measurements remains limited. Most previous studies have focused on a small number of parameters, frequently restricted to left ventricular function, and have often been conducted under controlled research conditions rather than within the context of a standardized clinical echocardiography laboratory in real-world practice (6).

In this context, the present study aimed to evaluate the reproducibility of a broad set of echocardiographic variables routinely used in clinical practice and representative of both left and right ventricular function in a heterogeneous population, including the simultaneous assessment of intraobserver and interobserver variability. In addition, we examined the agreement and precision of selected echocardiographic measurements relative to cardiac magnetic resonance in a standardized clinical echocardiography laboratory at a Latin American cardiovascular referral center.

## Methods

### Study population

Two cohorts were established for the analyses according to the following selection criteria. The inclusion of both healthy volunteers and patients undergoing clinical echocardiography allowed evaluation across a wide range of cardiac morphology and ventricular function. The clinical cohort consisted of consecutive adult patients referred for transthoracic echocardiography because of cardiovascular symptoms or as part of a routine cardiovascular evaluation. From this population, a sample of patients with two-dimensional images of sufficient quality for post-processing and repeated measurements was selected by simple random sampling.

The reference group consisted of healthy volunteers aged 18–65 years, with no history of cardiovascular disease or self-reported systemic illness, identified from an institutional database of blood donors.

### Image adquisition and postprocessing

Transthoracic echocardiographic studies were acquired using Philips EPIQ 7 and EPIQ CVx ultrasound systems (X5-1 transducer) by internationally certified cardiac sonographers (Registered Diagnostic Cardiac Sonographers, RDCS), in accordance with the recommendations of the American Society of Echocardiography (ASE) for comprehensive transthoracic echocardiography in adults (2).

The images were analyzed using TOMTEC-Arena Ultrasound Workspace software (TTA 2.4; TomTec Imaging Systems, Unterschleißheim, Germany). All studies were independently reviewed by 2 cardiologists with international certification and advanced training (Level III) in echocardiography.

Left-sided measurements included left ventricular end-diastolic volume (LVEDV), left ventricular end-systolic volume (LVESV), left ventricular ejection fraction (LVEF), left ventricular outflow tract diameter (LVOT diameter), left ventricular outflow tract velocity-time integral (LVOT VTI), and left atrial volume index (LAVI). Right-sided measurements included right ventricular basal diameter (RV basal diameter), right ventricular end-diastolic area (RVEDA), right ventricular end-systolic area (RVESA), right ventricular fractional area change (FAC), right atrial area (RA area), tricuspid annular plane systolic excursion (TAPSE), and tissue Doppler-derived tricuspid annular systolic velocity (TDI S’).

All patients included in the study underwent cardiac magnetic resonance imaging (CMR) on a 1.5-Tesla Philips Ingenia scanner (Philips Healthcare, Andover, Massachusetts) within 72 h of the echocardiographic examination. Standard cine images were acquired using balanced steady-state free precession sequences. Image analysis was performed offline using IntelliSpace Portal (Philips Healthcare). End-diastole and end-systole were defined as the frames showing the largest and smallest ventricular cavity area, respectively. Endocardial and epicardial contours were manually traced to derive ventricular volumes and left ventricular mass, with papillary muscles included as part of the compacted myocardium. The variables of interest were left ventricular end-diastolic volume (LVEDV), left ventricular end-systolic volume (LVESV), and left ventricular ejection fraction (LVEF).

To assess interobserver variability, two echocardiographers independently analyzed the studies while remaining blinded to the measurements performed by the other observer, thereby ensuring independence of the measurements. For the assessment of intraobserver variability, two observers repeated the analysis of a randomly selected subset of studies after a time interval of no less than one week, while remaining blinded to the results of the previous measurements.

### Statistical analysis

Continuous variables are presented as mean ± standard deviation or median (interquartile range), and categorical variables as absolute frequencies and percentages. Comparisons between healthy and diseased participants were performed using Welch's *t*-test for continuous variables with approximately normal distribution, the Mann–Whitney *U*-test for non-normal distributions, and the *χ*^2^ test or Fisher's exact test for categorical variables, as appropriate.

Interobserver reproducibility of echocardiographic measurements (Observer 1 vs. Observer 2) was quantified using the intraclass correlation coefficient (ICC) for absolute agreement, employing a two-way random-effects model with single measurements (ICC[2,1]). For each parameter, the standard error of measurement (SEM) and coefficient of variation (CV%) were calculated. The minimal detectable change at the 95% confidence level (MDC95) was derived using the formula: MDC95 = 1.96 × √2 × SEM.

Agreement was also evaluated using Bland–Altman analysis, reporting the mean bias (Observer 1–Observer 2) and the 95% limits of agreement (LoA). Bland–Altman plots were generated for LVEF and RV FAC.

For method comparison between echocardiography and CMR, agreement was assessed using ICC (2,1) for absolute agreement and Bland–Altman analysis, reporting the bias (Echo−CMR) and the 95% limits of agreement. Pearson correlation coefficients were reported only as complementary measures of association and were not used to infer agreement, which was assessed primarily by ICC and Bland–Altman analysis. All statistical tests were two-sided, and a *p*-value < 0.05 was considered statistically significant. Statistical analyses were performed in R (R Foundation for Statistical Computing) using RStudio (Posit Software, PBC; version 2026.01.0 + 392).

### Ethical considerations

For the reference group, all participants provided written informed consent for echocardiographic examination and use of the study data. The study was conducted in accordance with the principles of the Declaration of Helsinki and was approved by the institutional ethics committee within the framework of a broader study designed to assess normal echocardiographic values in a Colombian population.

For the clinical group, data were obtained from institutional records and were fully anonymized before analysis. No intervention was performed, and no diagnostic or therapeutic decisions were modified as part of the study. The study adhered to international ethical standards and, according to Colombian regulations, was classified as research without risk in accordance with Resolution 8,430 of 1993.

## Results

### Study population

A total of 63 participants were included in the analysis, including 20 individuals in the reference group and 43 in the clinical group. The median age of the overall cohort was 54 years (IQR 42–63), and 63% were male. Participants in the clinical group were older than those in the reference group, whereas sex distribution was similar between groups.

Compared with the reference group, participants in the clinical cohort showed larger LVEDV, LVESV and lower LVEF. Left atrial volume index was also higher in the clinical group. A similar pattern was observed for right-sided chambers, with larger RV basal diameter, RVEDA, RVESA, and RA area in the clinical group. In addition, right ventricular systolic function parameters were lower in the clinical cohort, including FAC, TAPSE, and TDI S’. A detailed comparison of baseline clinical and echocardiographic characteristics is presented in Table 1.

**Table 1:** Baseline clinical and echocardiographic characteristics by health status (healthy vs. Clinical).

Characteristic	Overall	Healthy	Clinical	*p* value
	*N* = 63^a^	*N* = 20^a^	*N* = 43^a^	
Age	54 [42, 63]	42 [31, 54]	57 [51, 64]	< 0.001
Gender				0.78129
Male	40 (63%)	12 (60%)	28 (65%)	0.78129
Female	23 (37%)	8 (40%)	15 (35%)	0.78129
LVEDV (mL)	107 [88, 152]	98 [87, 109]	137 [91, 194]	0.0188
LVESV (mL)	50 [35, 102]	37 [33, 42]	71 [38, 151]	< 0.001
LVEF (%)	56 [27, 62]	62 [61, 64]	41 [21, 56]	< 0.001
FAC (%)	39 [28, 43]	42 [39, 44]	34 [25, 42]	0.01542
TAPSE (mm)	20.3 [14.5, 23]	22 [19.4, 23]	15.5 [13, 22]	< 0.001
TDI-Ś(cm/s)	11 [9, 13]	12 [11, 14]	10.2 [8.4, 11.5]	< 0.001
RVBD (mm)	35 [32, 41]	34 [33, 35]	37 [32, 43]	0.03749
RVEDA (cm^2^)	20 [17, 26]	18 [15, 20]	22 [16, 30]	0.01531
RVESA (cm^2^)	12 [9, 17]	11 [9, 12]	14 [10, 22]	0.00566
RA area (cm^2^)	17 [13.5, 20.5]	14 [12, 17]	20 [16.0, 24.0]	< 0.001
LAVI (mL/m^2^)	31 [25, 41]	25 [22, 27]	35 [30, 63]	< 0.001
LVOT diameter (cm)	2.05 [1.97, 2.15]	2.04 [1.94, 2.12]	2.08 [1.98, 2.16]	0.21213
LVOT area (cm^2^)	3.3 [3.08, 3.65]	3.26 [2.96, 3.54]	3.41 [3.08, 3.67]	0.14575
LVOT PW (cm)	18.5 [15.7, 21.4]	21.27 [17.83, 23.08]	17.05 [13.5, 20]	0.00428
SV (mL by Simpson)	53 [43, 67]	62 [52, 70]	47.5 [38, 61]	0.00189
SV (mL by CE)	62 [50.5, 77]	63 [58, 80]	61 [48, 73]	0.23190

a Median [Q1, Q3]; *n* (%).

Continuous variables are presented as median [Q1, Q3] and categorical variables as *n* (%). *P* values compare the healthy and clinical groups using the Wilcoxon rank-sum test for continuous variables and Fisher's exact test for categorical variables.

LVEDV, indicates left ventricular end-diastolic volume; LVESV, left ventricular end-systolic volume; LVEF, left ventricular ejection fraction; FAC, fractional area change; TAPSE, tricuspid annular plane systolic excursion; TDI S’, tissue Doppler–derived tricuspid annular systolic velocity; RVBD, right ventricular basal diameter; RVEDA, right ventricular end-diastolic area; RVESA, right ventricular end-systolic area; RA area, right atrial area; LAVI, left atrial volume index; LVOT, left ventricular outflow tract; PW, pulsed-wave Doppler; SV, stroke volume; Simpson, biplane method of disks; and CE, continuity equation.

### Interobserver reproducibility

**Figure 1:**
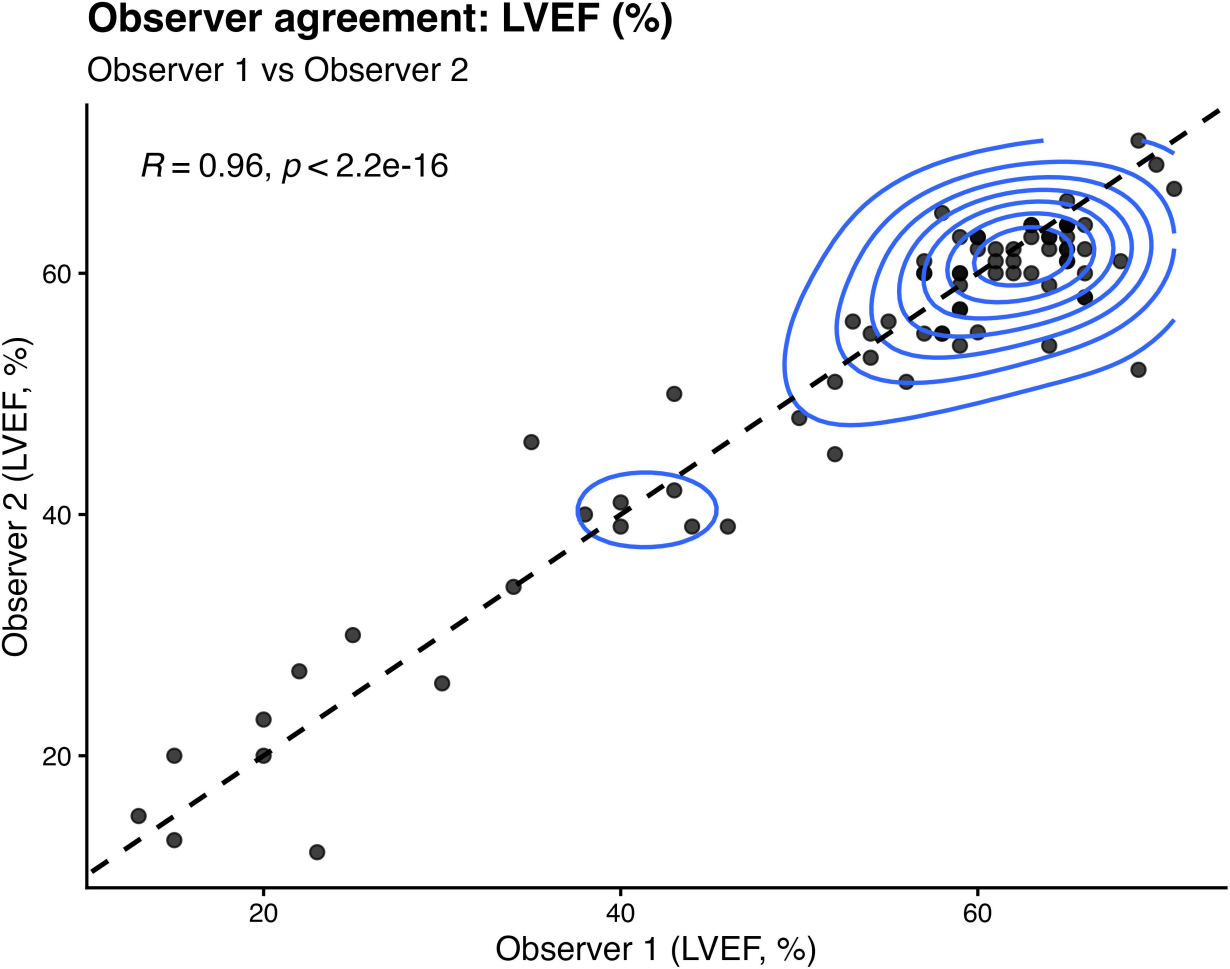
Interobserver agreement for left ventricular ejection fraction. Identity plot comparing LVEF measurements obtained by Observer 1 and Observer 2. The dashed line indicates the line of identity, and blue contours represent the density distribution of the observations. The close clustering of data points around the identity line reflects excellent interobserver agreement.

**Figure 2:**
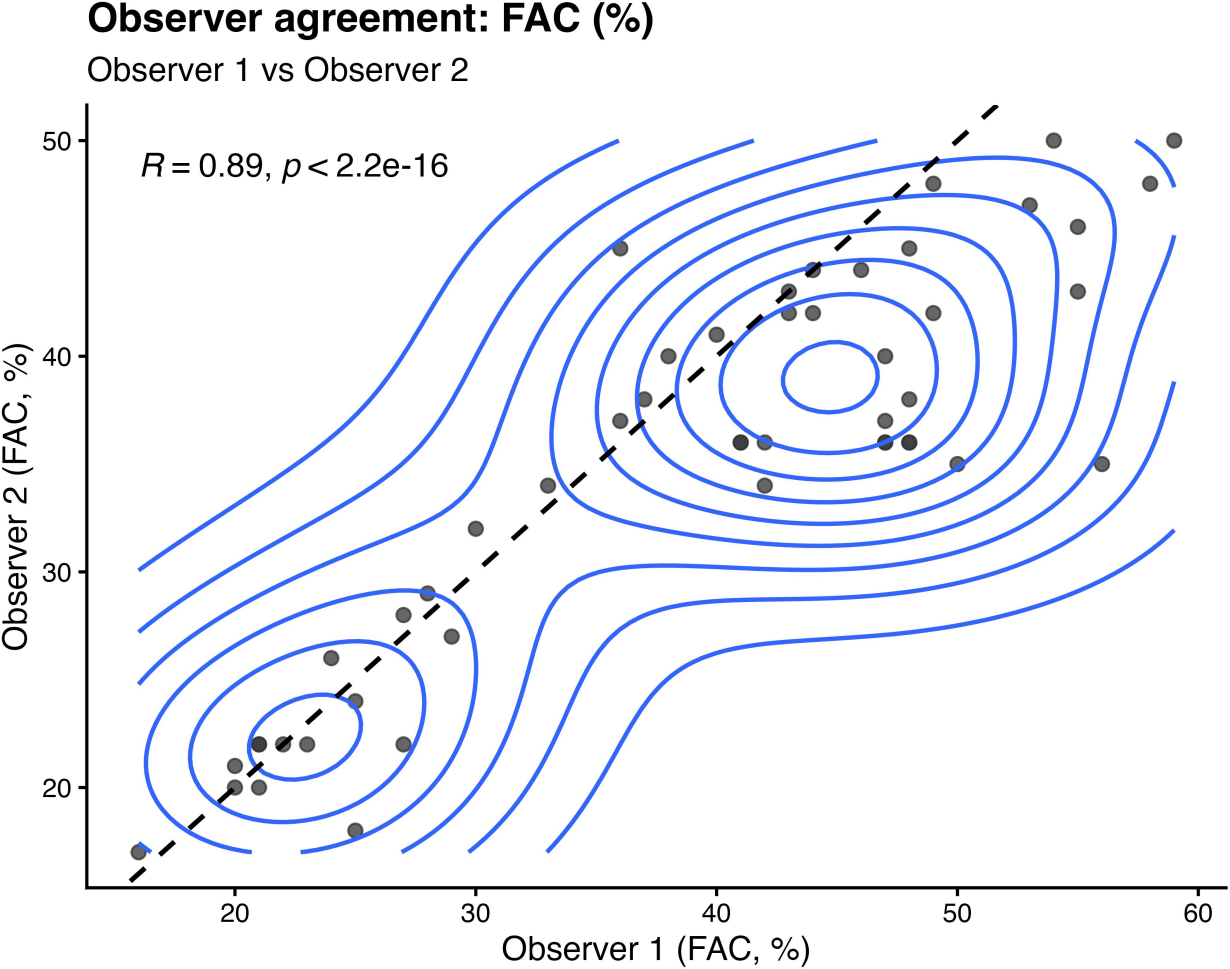
Interobserver agreement for fractional area change. Identity plot comparing FAC measurements obtained by Observer 1 and Observer 2. The dashed line indicates the line of identity, and blue contours represent the density distribution of the observations. Data points are more widely distributed around the identity line than for LVEF, indicating greater dispersion between observers.

Interobserver agreement between Observer 1 and Observer 2 is summarized in Table 2. Overall, reproducibility was excellent for left ventricular measurements and good to excellent for most right ventricular and atrial parameters. Left ventricular volumes showed near-perfect agreement, and LVEF also demonstrated excellent reproducibility, with a mean interobserver difference of 1.13 percentage points. The identity plot showed close clustering of LVEF measurements around the line of equality (Figure 1). LVOT PW Doppler VTI and RV basal diameter also showed excellent agreement. RV area measurements, TAPSE, TDI S’, FAC, and RA area remained in the good range, whereas LAVI showed excellent agreement. FAC measurements were more dispersed around the line of equality in the identity plot (Figure 2). Among all variables, LVOT diameter showed the lowest concordance. Stratified interobserver reproducibility analyses by health status are provided in Supplementary Table S1.

**Table 2:** Interobserver reproducibility.

Parameter	ICC (2,1)	ICC (95% CI)	Bias	CV (%)	SEM	MDC95
LV EDV (mL)	0.978	0.961–0.987	4.69	7.35	10.2	28.36
LV ESV (mL)	0.994	0.990–0.997	1.00	6.67	5.23	14.51
LVOT diameter (cm)	0.637	0.361–0.826	0.15	4.32	0.13	0.35
LVEF (%)	0.953	0.926–0.970	1.13	4.35	2.31	6.4
LVOT PW-Doppler VTI (cm)	0.939	0.855–0.975	0.67	3.09	0.77	2.13
RV basal diameter (mm)	0.916	0.857–0.951	−0.14	6.20	2.31	6.39
RV end-diastolic area (cm^2^)	0.870	0.786–0.923	−1.88	10.69	2.65	7.35
RV end-systolic area (cm^2^)	0.902	0.835–0.943	−1.94	12.24	2.19	6.07
FAC (%)	0.813	0.526–0.913	3.9	10.9	4.03	11.2
RA area (cm^2^)	0.841	0.736–0.906	−0.93	13.62	2.51	6.97
LAVI (mL/m^2^)	0.947	0.909–0.969	−1.91	10.39	3.89	10.78
TAPSE (mm)	0.836	0.682–0.919	0.53	13.30	2.28	6.33
TDI Ś (cm/s)	0.750	0.526–0.876	−0.57	11.30	1.18	3.27

Per-parameter analyses were performed using paired complete cases only. Intraclass correlation coefficients are reported as ICC(2,1), corresponding to a 2-way random-effects model for absolute agreement based on single measurements. Bias was defined as Observer 1 minus Observer 2. CV indicates coefficient of variation; SEM, standard error of measurement; and MDC95, minimal detectable change at the 95% confidence level.

### Bland–altman analysis

**Figure 3:**
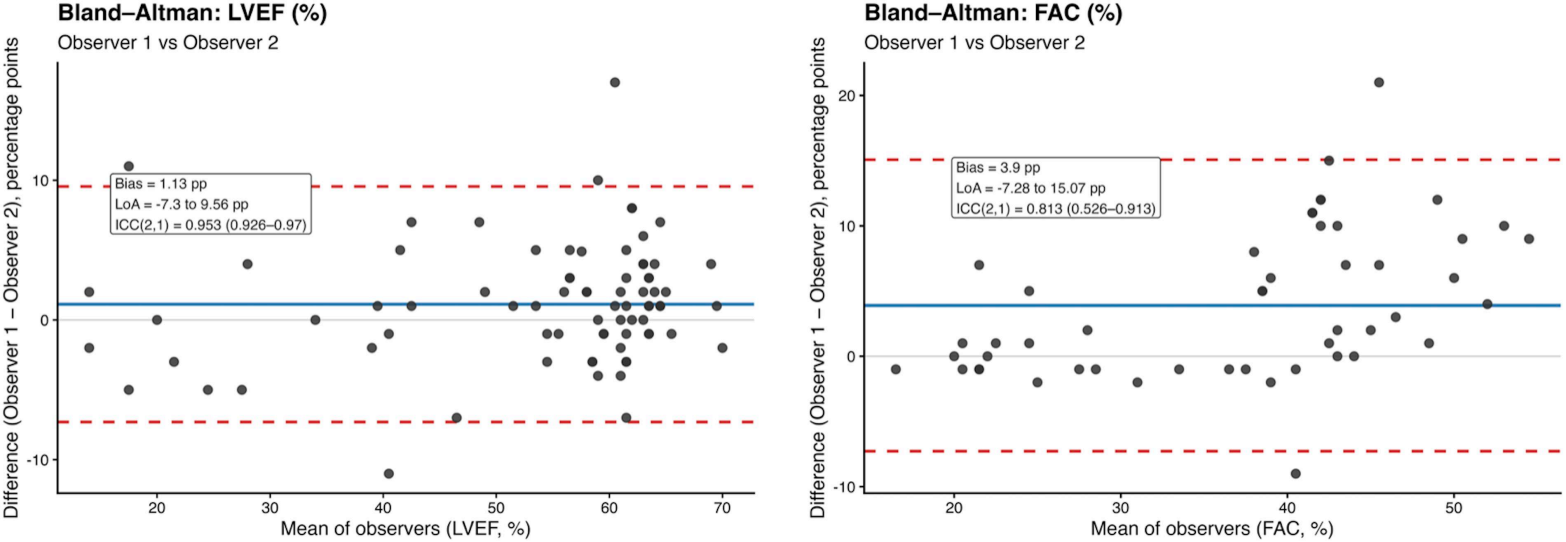
Bland–Altman plots for interobserver agreement in LVEF and FAC. Bland–Altman analysis comparing measurements obtained by Observer 1 and Observer 2 for left ventricular ejection fraction (LVEF, left panel) and fractional area change (FAC, right panel). The solid blue line represents the mean bias, and the dashed red lines indicate the 95% limits of agreement. LVEF showed a small mean interobserver difference with narrower limits of agreement, whereas FAC showed greater dispersion and wider limits of agreement.

Given the clinical importance of absolute agreement in indices of systolic function, Bland–Altman analyses were performed for LVEF and FAC (Figure 3).

For LVEF, the mean interobserver difference was small, with relatively narrow 95% limits of agreement and no evidence of proportional bias. In contrast, FAC showed wider limits of agreement and significant proportional bias, indicating that the magnitude of the discrepancy between observers varied across the measurement range.

### Intraobserver reproducibility of LVEF

In a subset of 17 studies, intraobserver reproducibility for LVEF was excellent for both readers. For Observer 1, repeated measurements showed an ICC of 0.961 (95% CI: 0.896–0.985), with a small mean bias of 0.53 percentage points and limits of agreement from −6.54 to 7.60. The corresponding measurement error was low, with a standard error of measurement (SEM) of 2.55, a coefficient of variation (CV) of 4.36%, and a minimal detectable change at the 95% confidence level (MDC95) of 7.07. Observer 2 demonstrated similarly excellent reproducibility, with an ICC of 0.984 (95% CI: 0.957–0.994), the same mean bias of 0.53 percentage points, and narrower limits of agreement (−3.55–4.61). Measurement error was even lower for Observer 2, with an SEM of 1.47, a CV of 2.53%, and an MDC95 of 4.08. Overall, these findings indicate excellent repeatability of LVEF measurements for both observers, with slightly higher stability and lower measurement noise for Observer 2.

### Comparison between echocardiography and cardiac magnetic resonance

**Figure 4:**
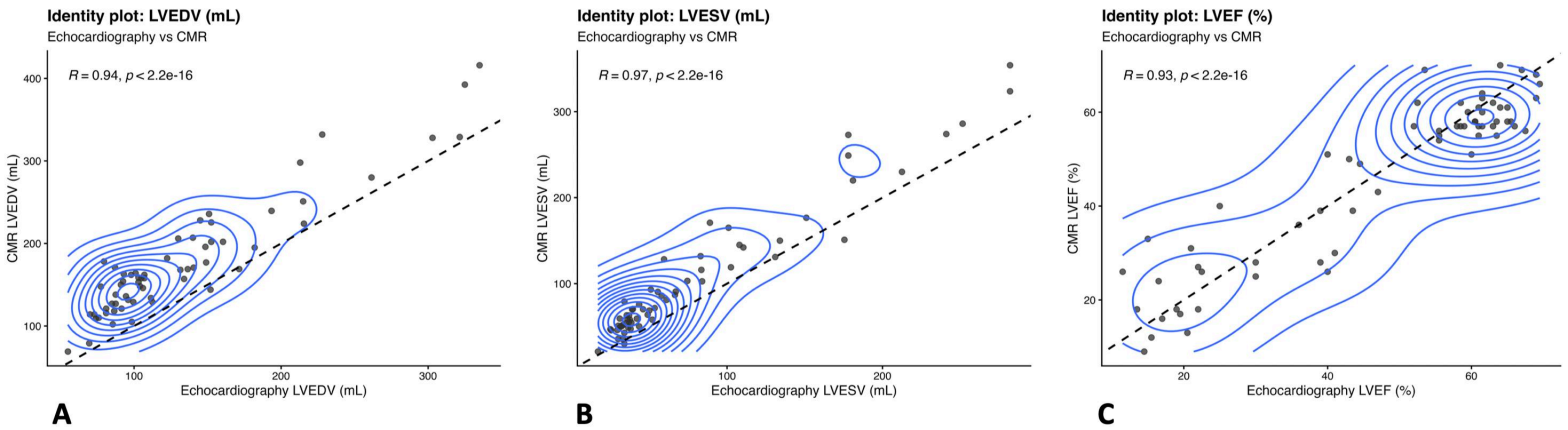
Identity plots for echocardiography vs. CMR agreement. Identity plots comparing echocardiographic and cardiac magnetic resonance (CMR) measurements of LVEDV **(A)**, LVESV **(B)**, and LVEF **(C)** The dashed line represents the line of identity, and blue contour lines indicate the density distribution of the observations. Although all parameters showed a strong linear association, LVEF measurements demonstrated closer agreement around the identity line than left ventricular volumes.

Paired comparisons between echocardiography and cardiac magnetic resonance imaging (CMR) demonstrated a strong linear association and good-to-excellent agreement for left ventricular volumes and LVEF (Table 3; Figure 4).

**Table 3:** Echocardiography vs. CMR agreement.

Parameter	Pearson r	*p*	ICC (2,1)	ICC 95% CI	CCC	Bias
LVEDV (mL)	0.937	<0.001	0.775	−0.055–0.935	0.772	−44.3
LVESV (mL)	0.969	<0.001	0.901	0.237–0.970	0.899	−26.4
LVEF (%)	0.931	<0.001	0.930	0.887–0.957	0.929	0.63

Per-parameter analyses were performed using paired complete cases only. Intraclass correlation coefficients are reported as ICC(2,1), corresponding to a 2-way random-effects model for absolute agreement based on single measurements. Bias was defined as echocardiography minus CMR. Limits of agreement and proportional-bias *P* values were derived from Bland–Altman analysis. CMR indicates cardiac magnetic resonance; LVEDV, left ventricular end-diastolic volume; LVESV, left ventricular end-systolic volume; LVEF, left ventricular ejection fraction; ICC, intraclass correlation coefficient; CCC, Lin's concordance correlation coefficient; and LoA, limits of agreement.

**Figure 5:**
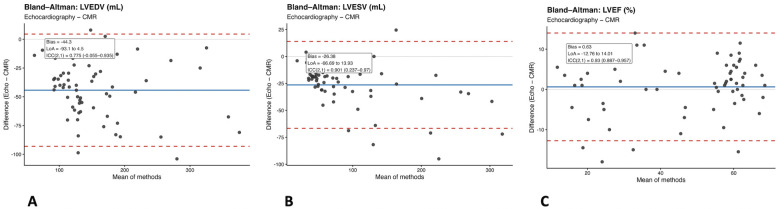
Bland–Altman plots for agreement between echocardiography and CMR. Bland–Altman plots comparing echocardiographic and cardiac magnetic resonance (CMR) measurements of left ventricular end-diastolic volume (LVEDV, panel **A**), left ventricular end-systolic volume (LVESV, panel **B**), and left ventricular ejection fraction (LVEF, panel **C**). The solid blue line represents the mean bias, and the dashed red lines indicate the 95% limits of agreement. Echocardiography systematically underestimated left ventricular volumes compared with CMR, whereas the mean bias for LVEF was minimal.

Both LVEDV and LVESV showed very high correlations and moderate-to-excellent agreement between modalities. However, echocardiography systematically underestimated ventricular volumes compared with CMR, as demonstrated by the Bland–Altman analyses (Figure 5).

Despite this volumetric discrepancy, LVEF showed excellent agreement between the two imaging modalities and minimal bias, suggesting that the estimation of global systolic function remains consistent even when differences in absolute ventricular volumes are present.

## Discussion

In this study, conducted in a standardized clinical echocardiography laboratory at a Latin American cardiovascular referral center, routine two-dimensional echocardiographic measurements showed overall good reproducibility, particularly for key left ventricular parameters. Greater variability was observed in measurements that are more dependent on image quality and tracing approach, especially FAC and LVOT diameter. As expected, echocardiography systematically underestimated left ventricular volumes compared with CMR, confirming that both techniques should not be considered interchangeable for volumetric quantification (7, 8). Nevertheless, agreement for LVEF was excellent and bias was minimal, supporting the consistency of this parameter across both modalities despite differences in the underlying measurement methodology.

The assessment of the reproducibility of echocardiographic measurements is a fundamental component of quality assurance in high-standard echocardiography laboratories. Moreover, reproducibility should be evaluated longitudinally and at regular intervals, with structured feedback and ongoing education to optimize image acquisition and standardize measurement protocols (9, 10).

Although direct comparisons with other laboratories are limited, prior studies have consistently shown high reproducibility for conventional echocardiographic measurements of left ventricular volumes and LVEF (10, 11). In contrast, reproducibility data for other routine two-dimensional measurements remain more limited, and these parameters are less often evaluated together within a single report. In our study, the principal indices used for the assessment of left ventricular function showed very high reproducibility (ICC > 0.9), supporting the robustness, precision, and reliability of these measurements between observers. LVESV measurements were slightly more stable than LVEDV. Stratified analyses by health status (Supplementary Table S1) showed some variability in subgroup-specific ICC estimates, particularly in the healthy subgroup, which is consistent with the narrower physiological range of values in smaller and more homogeneous samples rather than with a different overall pattern of measurement performance. The main potential sources of variability in volume measurements may be related to endocardial border delineation during diastole, the frame selected for measurement, the presence of more prominent myocardial trabeculations during this phase of the cardiac cycle, and, to some extent, apical foreshortening (1, 12, 13).

Regarding LVEF estimation, prior studies have generally shown good interobserver agreement. For example, Lennell et al. (14), in a cohort of patients with acute coronary syndrome, reported an interobserver ICC of 0.87 (95% CI: 0.82–0.92), which was slightly lower than that observed in our study. In our cohort, LVEF showed excellent reproducibility at the group level, with a mean interobserver difference of only +1.13 percentage points, indicating a small systematic bias and slight overestimation by one observer relative to the other. No relevant proportional bias was identified, as the discrepancy between measurements did not increase systematically across the range of LVEF values. Although the limits of agreement (−7.30 to +9.56 percentage points) indicate that clinically meaningful differences may still occur in individual patients, the combination of minimal mean bias and absence of proportional bias supports excellent overall reproducibility.

These findings are particularly relevant for the interpretation of serial LVEF measurements in clinical practice. In our laboratory, a change of approximately 7 percentage points exceeds expected interobserver variability and is therefore more likely to reflect a true biological change rather than measurement noise alone. This is especially important when LVEF values are close to clinically relevant decision thresholds, such as those used for heart failure classification, device therapy, or treatment selection. In such situations, small differences should be interpreted cautiously. When a measured change approaches or crosses a management threshold, confirmation with a repeat standardized measurement, ideally including a second observer, may improve confidence in the reported value and support more robust clinical decision-making.

With regard to the relationship between echocardiographic and cardiac magnetic resonance measurements of ventricular volumes and LVEF, a good linear correlation was observed between both imaging modalities. However, analysis of absolute agreement showed a different pattern, with good to excellent agreement for LVESV and moderate to good agreement for LVEDV. These findings are consistent with prior multimodality evidence showing that echocardiography and CMR should not be considered interchangeable for left ventricular volumetric quantification, even when the overall association between techniques is strong. In the meta-analysis by Rigolli et al. (15), 2-dimensional echocardiography systematically underestimated left ventricular volumes compared with CMR, with a mean bias of approximately −33 mL for LVEDV and −16 mL for LVESV, whereas differences in LVEF were minimal; this pattern improved with 3-dimensional echocardiography but was not completely eliminated. In our cohort, we observed the same direction of effect, with echocardiography underestimating LVEDV and LVESV by −44.3 mL and −26.4 mL, respectively, while agreement for LVEF remained excellent, with an ICC of 0.930, a CCC of 0.929, and a negligible bias of only +0.63 percentage points. From a practical perspective, this comparison is particularly illustrative: ventricular volumes are more sensitive to differences in image acquisition, endocardial border definition, and geometric assumptions, whereas LVEF appears to be more stable across modalities because it reflects a ratio rather than an absolute chamber measurement. Taken together, these findings support the use of echocardiography as a robust tool for LVEF assessment in routine practice, while reinforcing the need for caution when echocardiographic left ventricular volumes are interpreted alongside CMR values or used interchangeably during longitudinal follow-up.

With respect to right ventricular measurements, including basal diameter, end-diastolic and end-systolic areas, FAC, and the longitudinal indices TAPSE and TDI S’, reproducibility in our study was overall good. Although not as strong as that observed for left ventricular volumes and systolic function, these results still support the use of these parameters for routine echocardiographic assessment of right ventricular morphology and function. The greater variability of right ventricular measurements is biologically and technically plausible, given the chamber's anatomical complexity, irregular geometry, and the inability to capture all of its components within a single two-dimensional plane (16, 17). Previous studies have examined these indices mainly in terms of feasibility, correlation, or clinical validation, whereas data on their comparative reproducibility within a standardized workflow remain limited. In this regard, our findings differ from those of Pagnoni et al. (18), who reported substantially higher reproducibility for TDI S’ than for TAPSE in a highly standardized screening cohort of patients with systemic sclerosis and preserved right ventricular function. In contrast, in our cohort, TAPSE showed better reproducibility than TDI S’, and TAPSE agreement was also markedly higher than that reported in their study. These findings suggest that the relative performance of longitudinal right ventricular indices depends on the clinical setting, image acquisition, and measurement workflow.

Among the right ventricular indices, the proportional bias observed for FAC deserves particular attention because it suggests that interobserver discrepancies increase across the measurement range rather than remaining constant. FAC is particularly susceptible to variability because it requires manual delineation of both the end-diastolic and end-systolic right ventricular endocardial borders, making it especially sensitive to differences in identifying the thin right ventricular free wall, consistently excluding trabeculations from the cavity contour, and accurately defining the hinge points of the tricuspid annulus (17, 19). Because FAC is calculated from the relative change between end-diastolic and end-systolic cavity areas, even small inconsistencies in endocardial tracing may be amplified in the final measurement, providing a plausible explanation for the proportional bias observed in our study. From a practical perspective, reproducibility may be improved by acquiring a true RV-focused apical four-chamber view to minimize apical foreshortening, optimize visualization of the right ventricular free wall and apex, apply standardized tracing criteria for endocardial border delineation at both end-diastole and end-systole, and consistently exclude trabeculations from the endocardial contour (17).

One of the parameters most susceptible to lower reproducibility is LVOT diameter. This has important clinical implications in echocardiographic practice, including the estimation of aortic valve area, calculation of cardiac index, and volumetric assessment in valvular heart disease (16, 19). Because LVOT area is derived by squaring the measured diameter, even small measurement errors may be amplified when the continuity equation is applied (20). This variability likely reflects the strong dependence of LVOT diameter on the acquisition plane, the phase of the cardiac cycle, and the precise delineation of the endocardial borders. As reported by Guzzetti et al. (20), LVOT diameter decreases progressively when measured more distally from the annulus, underscoring the nonuniform geometry of the LVOT. In our study, LVOT diameter showed the lowest reproducibility among all evaluated parameters, highlighting the need for continued efforts to improve standardization of its measurement.

Overall, our findings suggest that routine two-dimensional echocardiographic measurements showed overall good reproducibility in this clinical laboratory setting, where studies were acquired and analyzed under standardized routine workflows by trained personnel. This is particularly relevant in contemporary echocardiography laboratories, where measurement reliability depends not only on the intrinsic properties of each parameter, but also on the consistency of image acquisition and operator training. At the same time, our results reaffirm the well-recognized challenge of right ventricular assessment, in which greater anatomical complexity and technical dependency continue to translate into higher variability than that observed for the left ventricle. These findings also highlight the practical relevance of standardized acquisition, measurement consistency, and quality control in echocardiographic laboratories.

## Limitations

Our study has several limitations. First, the sample size was relatively small, which may have limited the precision of some estimates, particularly for parameters with greater variability. In addition, the healthy reference group was younger than the clinical cohort. Although this reflects the characteristics of the populations from which each cohort was recruited, age-related differences in cardiac geometry and image quality may have influenced reproducibility estimates and cannot be completely excluded. No formal *a priori* sample size calculation was performed. However, in ICC-based reproducibility studies, sample size requirements depend primarily on the expected reliability coefficient, number of raters, and desired confidence interval width rather than on conventional effect-size based power calculations. Under this framework, assuming an expected ICC of approximately 0.85 and a desired 95% confidence interval width of 0.20, our sample size can be considered adequate for the primary reproducibility objective of the study (21). Accordingly, the main consequence of the sample size in our study is reduced precision for some estimates rather than invalidation of the overall reproducibility analysis. In addition, because reproducibility metrics such as the ICC depend on the range and distribution of the underlying values, the heterogeneous composition of the cohort may have influenced agreement estimates, whereas the smaller healthy subgroup, with its narrower physiological range, likely yielded less stable subgroup-specific coefficients. To address this issue, we added stratified reproducibility analyses by health status as Supplementary Table S1; however, these subgroup-specific estimates should be interpreted cautiously, particularly in the healthy subgroup, given its limited sample size. In addition, intraobserver reproducibility was evaluated only for LVEF. Although interobserver agreement represented the primary objective of the present study, repeatability of right ventricular and LVOT measurements within the same observer was not systematically assessed and should be addressed in future investigations. Second, image quality remains an important source of selection bias in reproducibility studies. Measurements were not performed or reported when technical quality was inadequate for reliable analysis, in keeping with routine echocardiographic practice. Although the overall feasibility of image acquisition for the selected parameters was high in our cohort and poor image quality did not represent a major source of attrition in the analyzed sample, our findings still reflect reproducibility under conditions of adequate technical feasibility and may not fully represent populations with a higher prevalence of suboptimal imaging. Third, measurements were performed using conventional two-dimensional echocardiography and M-mode, without incorporating three-dimensional echocardiography or newer artificial intelligence–based tools that, under selected technical conditions, may improve measurement accuracy and reproducibility. Nevertheless, this approach reflects the techniques most commonly used in daily clinical practice and therefore preserves the practical applicability of our findings. Finally, as with any study evaluating an operator-dependent imaging modality, measurement quality is influenced by observer expertise. Because all measurements were performed by experienced readers in a real-world echocardiography laboratory at a Latin American cardiovascular referral center, using standardized workflows, the reproducibility observed in our study may not be directly generalizable to laboratories with different levels of experience, training, or quality control, although it does represent performance under optimized real-world conditions.

## Conclusion

In this study, routine echocardiographic parameters used for the assessment of left and right ventricular systolic function showed overall good reproducibility, with the most robust performance observed for conventional left ventricular measurements. Greater variability was identified in selected right ventricular indices and in LVOT diameter, underscoring the need for careful acquisition and standardized measurement protocols for these parameters. When compared with cardiac magnetic resonance, echocardiography systematically underestimated left ventricular volumes but showed excellent agreement for LVEF, supporting its reliability for the assessment of global systolic function in daily practice. Although reproducibility was excellent at the population level, clinicians should recognize that individual LVEF measurements may still differ by clinically meaningful amounts. Consequently, small serial changes in LVEF should be interpreted cautiously, particularly when values are close to clinically relevant decision thresholds, and should ideally be obtained and interpreted using standardized acquisition and measurement protocols. Overall, these findings support the practical reliability of routine echocardiographic measurements in this real-world clinical laboratory setting and highlight the value of reproducibility assessment as an integral component of echocardiography laboratory quality assurance.

## Data Availability

The raw data supporting the conclusions of this article will be made available by the authors, without undue reservation.
