# Supplementary material for: Routine echocardiographic measurements in clinical practice: reproducibility of left and right ventricular indices and agreement with cardiac magnetic resonance
Source: Front Cardiovasc Med. 2026 Aug 5;13:1843290. doi: 10.3389/fcvm.2026.1843290 (PMC13485716; doi:10.3389/fcvm.2026.1843290)
Supplement: Supplementary file 1 [file Table1.docx]

**Supplementary Table S1. Interobserver reproducibility metrics by health-status group**

| **Group** | **Parameter** | **N paired** | **ICC (2,1)** | **ICC lower** | **ICC upper** |
| --- | --- | --- | --- | --- | --- |
| Healthy | LV EDV (mL) | 20 | 0.922 | 0.81 | 0.97 |
| Healthy | LV ESV (mL) | 20 | 0.784 | 0.53 | 0.91 |
| Healthy | LVOT diameter (cm) | 20 | 0.637 | -0.04 | 0.88 |
| Healthy | LVEF (%) | 20 | 0.329 | -0.07 | 0.65 |
| Healthy | LVOT PW-Doppler VTI (cm) | 20 | 0.939 | 0.75 | 0.98 |
| Healthy | RV basal diameter (mm) | 20 | 0.515 | 0.1 | 0.78 |
| Healthy | RV end-diastolic area (cm²) | 20 | 0.573 | 0.1 | 0.82 |
| Healthy | RV end-systolic area (cm²) | 20 | 0.396 | -0.08 | 0.72 |
| Healthy | FAC (%) | 20 | 0.15 | -0.15 | 0.49 |
| Healthy | RA area (cm²) | 20 | 0.562 | 0.09 | 0.81 |
| Healthy | LAVI (mL/m²) | 20 | 0.583 | 0.21 | 0.81 |
| Clinical | LV EDV (mL) | 42 | 0.978 | 0.95 | 0.99 |
| Clinical | LV ESV (mL) | 42 | 0.993 | 0.99 | 1 |
| Clinical | LVOT diameter (cm) | 43 | 0.527 | 0.15 | 0.74 |
| Clinical | LVEF (%) | 43 | 0.965 | 0.94 | 0.98 |
| Clinical | LVOT PW-Doppler VTI (cm) | 42 | 0.857 | 0.74 | 0.92 |
| Clinical | RV basal diameter (mm) | 43 | 0.88 | 0.79 | 0.93 |
| Clinical | RV end-diastolic area (cm²) | 43 | 0.905 | 0.82 | 0.95 |
| Clinical | RV end-systolic area (cm²) | 41 | 0.929 | 0.81 | 0.97 |
| Clinical | FAC (%) | 41 | 0.818 | 0.6 | 0.91 |
| Clinical | RA area (cm²) | 31 | 0.832 | 0.68 | 0.92 |
| Clinical | LAVI (mL/m²) | 31 | 0.94 | 0.87 | 0.97 |

Per-parameter analyses were performed using paired complete cases only within each health-status subgroup. Intraclass correlation coefficients are reported as ICC(2,1), corresponding to a 2-way random-effects model for absolute agreement based on single measurements.
